# Using simulation scenarios and a debriefing structure to promote feedback skills among interprofessional team members in clinical practice

**DOI:** 10.1186/s41077-024-00303-5

**Published:** 2024-09-18

**Authors:** Bodil Thorsager Svendsen, Lene Funck Petersen, Anders Skjelsager, Anne Lippert, Doris Østergaard

**Affiliations:** 1 Department of Anaesthesia and Intensive Care, Herlev Gentofte University Hospital, Hellerup, Denmark; 2 Copenhagen Academy for Medical Education and Simulation CAMES, Herlev Hospital, Capital Region of Denmark, Borgmester Ib Juuls Vej 1, Opgang 1, etage 25, 2730 Herlev, Denmark; 3 Department of Clinical Medicine, University of Copenhagen, Copenhagen, Denmark

## Abstract

**Background:**

Team reflexivity and peer feedback in daily clinical work can improve patient safety. However, teams do not always engage in reflection after patient care. A reason could be that team members may lack skills in engaging in team reflection. This study explores the use of interprofessional team-based simulations to encourage and equip teams for reflective conversations in the real-world clinical practice.

**Methods:**

This was a prospective, explorative study of team members’ perceptions of the use of in situ simulation-based scenarios with critically ill patient cases to train team-based reflections and peer feedback. The study took place in two neurological wards. Prior to the intervention, a 1-day observation in each ward and semi-structured short interviews with physicians and nurses were conducted.

**Results:**

A total of 94 staff members, 57 nurses, 8 nurse assistants and 29 physicians participated in the in situ simulation scenarios. All team members showed appreciation of the safe learning environment. The authors found that the simulations and the debriefing structure provided an opportunity for training of team reflexivity and feedback. The team members evaluated the simulation-based training very positively, and their initial reaction indicated that they found peer feedback useful for the individual and the team. This approach allowed them to reflect on their own clinical practice.

**Conclusion:**

The simulation-based training scenarios and the debriefing structure promoted team members’ team reflexivity and peer feedback skills. The method is feasible and could be used in other specialties and situations.

The team members’ reactions to feedback were positive, and based on their reflections, there is a potential to increase both individual and team skills as well as improve patient treatment.

## Introduction

Feedback is one of the most important factors for adult learning [1, 2]. It is used mainly as an important part of clinical training for novices but could also provide an opportunity for staff to support life-long learning. In a recent report from the Lancet Commission, learning for life was mentioned in relation to the demands of healthcare professionals’ continuous development [3]. Metacognition and the ability to reflect on ones’ work is mentioned as important competencies. In addition, interprofessional activities and training of teams were mentioned as important [3] as competence in a complex clinical setting is a collective phenomenon [4, 5]. Clinical teams perform better when they engage in team reflection and peer feedback [6] However, team members may lack the skills to engage in team reflection. Previously, most educational activities were mono-professional activities, such as congresses or courses. Now, a paradigm shift is seen in favour of workplace-based learning, but as it is difficult to be objective about one’s own competencies, input from others is essential [7]. Team reflexivity and peer feedback could strengthen dialogue and learning in the context of daily clinical work. In residency training, the R2C2 (relationship, reaction, content and coaching) model has been successfully used [8]. However, knowledge on how to train staff members to provide feedback and how to implement this feedback in busy clinical wards is limited. A conceptual framework for team reflexivity in health care has been described by Schmutz and Eppich [5]. The authors suggest that team reflexivity should occur before patient care, during active care and after patient care.

Simulation-based training of multiprofessional teams conducted either in simulation centres or in situ is becoming increasingly widespread. Debriefing after a simulation is an opportunity for participants to discuss and reflect on performance [9, 10]. Despite the benefit of debriefing for individuals and teams, the method itself is seldom brought back to the workplace [11, 12]. The question is why? One explanation could be the lack of psychological safety in interactions between professions. Learning is a process in which a team seeks knowledge from team members, provides feedback and reflects and discusses results, including errors or gaps in performance [13]. For learning to take place, team members must feel psychologically safe [13].

We sought to explore how interprofessional, in situ simulations with self-debriefing might translate to team-based reflections and peer feedback in real clinical practice.

## Aim

The aim of this study was to explore how the use of team-based in situ simulation scenarios with self-debriefing (using a debriefing structure) could promote team reflexivity and peer feedback skills in clinical teams. We aimed to evaluate the team members’ perception of the feasibility and impact of the training.

## Methods

### Context and participants

The study took place in two in-patient wards, which are part of the neurological department, in a large university hospital in the Capital Region of Denmark. The physicians work in both wards, whereas each nursing staff member is dedicated to one ward only. The department admits patients with multiple diagnoses, including many acute admissions, e.g. of patients diagnosed with stroke.

The staff expressed a need for improvement in interprofessional collaboration in the handling of critically ill patients. The head of the department contacted our simulation centre, Copenhagen Academy for Medical Education and Simulation (CAMES), to ask for help and to initiate a project. Local representatives of physicians and nurses from both wards were appointed, and a plan for how to identify problems and agree on shared training needs was established.

### Preintervention activities and data sampling

We followed Kern’s six-step approach for curriculum development that includes problem identification, targeted needs assessment, goals and objectives, educational strategies, implementation and evaluation [14]. Prior to the intervention, one of the authors (LFP) conducted a 1-day observation in each ward and semi-structured short interviews with physicians and nurses (8 in total) to understand “how work is done”. LFP is an intensive care nurse with experience with critically ill patients and teamwork. LFP has previously participated in several projects involving observations. The main findings were related to collaboration between professionals in critical situations. The nurses lacked the possibility to talk about the situation afterwards—to talk about what they did well and if they could have done things differently. They were curious about the physicians’ views of the situation and what their expectations were. The nurses mentioned that it takes time for a newly graduated nurse to obtain experience with critically ill patients, as their clinical training is limited. Good collaboration between nurses and nurse assistants was mentioned. Based on the information gained from the observations and interviews, a workshop was planned.

**Fig. 1:**
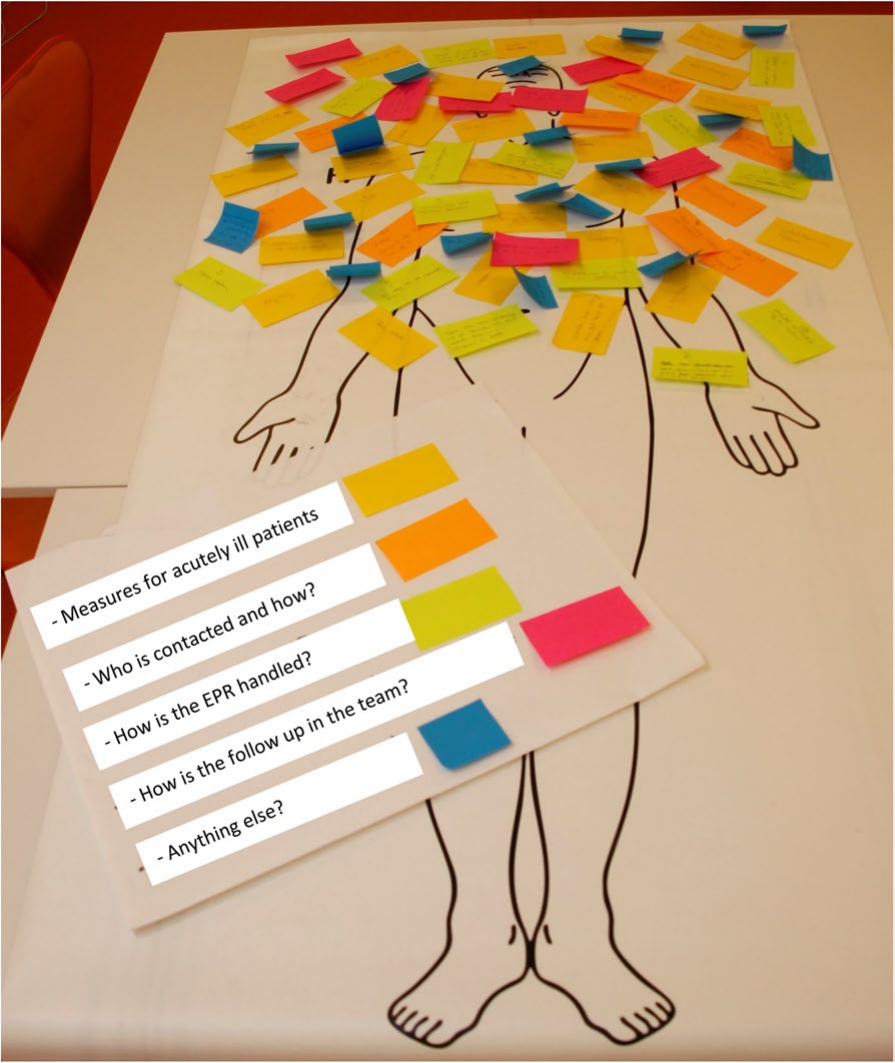
The table-top simulation model used in the workshop

Two of the authors (LFP and BTS) conducted a workshop involving 5 physicians and nurses from the wards. BT is an anaesthesiologist with experience in handling critically ill patients. The purpose of the workshop was to understand the approach taken to critically ill patients and the tasks the team performed during and after the encounter. A simplified table-top simulation, with a drawing of a patient, was used to illustrate the patient’s situation. The participants were asked approximately (1) their tasks related to a deteriorating patient, such as vital signs (yellow label), (2) who is called and how (orange label), (3) how is it recorded in the electronic patient record (green label), (4) how and who is responsible for the follow-up after the situation (red label) and (5) what we as facilitators may have forgotten in this process (blue label). See Fig. 1. The data from the labels were transcribed, and the results were discussed with the local representatives and the heads of department. In particular, the red-labelled data were rich, and it was clear that there was room for improvement in the follow-up after critical situations. Some of the nurses expressed that they were afraid to call junior physicians, while others thought that the physician knew all the patients and would solve all the problems. One nurse mentioned that junior physicians should be better at explaining their priorities. It was also noted that the development of the situation depends on who is part of it, which, the participants thought, should not be the case. The workshop participants expressed concern that the team would split up immediately after taking care of a critically ill patient, without a reflection on what could be improved. All agreed that feedback after a critical situation is needed and that it should be interprofessional. This approach could increase competence and improve patient safety.

Based on the findings of the workshop, the intervention was planned. It was decided to train staff members team reflexivity and interprofessional, peer feedback skills immediately after encountering acutely ill patients by using in situ simulation scenarios followed by a debriefing. The development was based on conceptual frameworks: Kolb’s learning cycle, reflexivity theory and peer feedback.

### The development of the intervention

Posters and flyers addressed to all staff members were developed to prepare them for the intervention. The authors attended meetings in the wards to provide information about the training intervention.

We recorded a video illustrating how an in situ simulation could be conducted to prepare them for the scenario and develop a safe learning environment. Finally, a video illustrating how to handle a critically ill patient was developed and made available to all staff members to prepare them for the initial treatment of a critically ill patient. The video illustrates the correct use of an ABCDE approach for a critically ill patient and the Danish modified version of the SBAR structured communication tool, an ISBAR where an I for identification is included.

Scenarios illustrating a critically ill patient were developed based on real patient events from the ward. The scenarios were designed to encompass a familiar situation in the 2 wards. In one of the wards, a patient with known haemorrhagic stroke developed an aspiration pneumonia and deteriorated; in the second ward, the patient suffered from an increased frequency of epileptic seizures and eventually status epilepticus. A learning manual describing the learning objectives, the relevant patient information, the development of the case and additional blood test results and the description of the roles and information to be given to the team members were made available to trained facilitators from CAMES. The learning objectives were read aloud to the team before the simulation began. The main objectives of the scenarios were to support team members, train team skills and use the ABCDE and ISBAR structures.

### The intervention

We planned to train four teams on each of the eight training days, which took place over a 5-month period. The agenda for the training days is shown in Table 1. Each team had 90 min of training time. The teams were planned to include 1–2 physicians and 3–4 nurses or nurse assistants. The team was introduced to peer feedback and how to praise what went well and how to ask questions such as “I saw you did… I wonder why did ….”. The simulations took place in the neurological ward itself to increase the realism of actions in the simulations and to diminish the time away from clinical work. We placed a simulator and monitor in a bed, which we brought to the ward. The clinical team used the equipment and medications available in the ward. Two facilitators from CAMES conducted the scenario and briefly summarised the scenario to handle any immediate questions. In contrast to usual simulation-based training, there was not a facilitator lead debriefing of the scenario.

**Table 1:** Agenda for training sessions

Welcome and introduction	15 min
Introduction to the debriefing structure and feedback	15 min
Simulation scenario	15 min
“Wrap up” – questions in relation to the scenario	5 min
Feedback training	30 min
Team members take home messages	10 min

After the scenario, the participants themselves followed a debriefing structure consisting of a description, an analysis and an application phase to provide feedback to each other [15, 16]. First, the team leader (the physician) led a short description phase, where the team members briefly described the clinical situation to obtain a common picture of the situation. Second, in the analysis phase, the team members mentioned what a given team member did well and why. The team members would then ask questions about things that another team member did, where they wondered why or were curious about their decision, to obtain a better understanding of the frames behind the actions. Third, in the application phase, every individual team member verbalised his or her own learning points that could be taken back into clinical practice. This step provides a window of opportunity for participants to commit to applying the learning from the simulation to their everyday work.

### Data collection

We collected three types of data from the team members: (1) their individual learning objectives after their participation in the scenario and the debriefing, (2) their perception of in situ simulation and a debriefing structure to train team reflexivity and peer feedback and (3) their satisfaction with the session.

The team members wrote their individual learning points on paper, which were collected and assembled in a spreadsheet and categorised by the research team using thematic content analysis [17, 18]. After familiarising the authors with the data, initial codes were generated by LFP and BTS. The codes were discussed, and themes were identified by all the authors. We realised that the themes were related primarily to social and cognitive skills, and we then chose to use the Danish Anaesthesia Non-Technical skills framework (ANTS.dk) [19] to deductively categorise the data.

Each participant was asked to write down their opinion and experience with in situ simulation and a debriefing structure as a method to improve interprofessional teamwork and peer feedback. The data were recorded in a spreadsheet and organised into themes as described above.

A questionnaire focusing on four statements with open space for comments was used for evaluating participants’ immediate satisfaction with the session. The statements were as follows: (1) I found the situation meaningful, (2) I perceived the debriefing situation as safe, (3) the debriefing structure gave me the opportunity to say, what I found most important, and (4) the debriefing situation made me reflect on my clinical practice. A Likert scale ranging from 0 to 5 (where 5 is strongly agree) was used. The questionnaire was given to all team members after the sessions. In addition, we asked the team members to complete the questionnaire again after clinical situations in the following weeks.

## Results

A total of 94 staff members, 57 nurses, 8 nurse assistants and 29 physicians participated in the in situ simulation scenarios. The number of participants in each of the scenarios varied from 3 to 6.

The total number of learning points verbalised after the feedback session was 192. The learning points illustrated the insights of individual team members and their intended actions for their clinical work. In Table 2, examples of the expressed learning points are shown as well as the overarching categories and elements. The percentages of verbalised learning objectives in the 4 ANTS.dk categories were teamwork (52%), leadership (18%), situation awareness (17%) and decision-making (13%). They mentioned the importance of sharing information to obtain a shared mental model of the situation and of speaking loudly and clearly. The use of “sum ups” of the situation was found to be helpful. They also talked about how team members could best support each other and facilitate others’ performance of the tasks. The team members appreciated structured tools such as the use of an ABCDE approach to the patient and the use of ISBAR to structure communication.

**Table 2:** Team member reflections and learning points for future clinical work

Non-technical skills category	Non-technical skills elements	Citations
**Situation awareness**	**Gathering information**	“Ask if something has happened while I was out of the room”
	**Anticipate and think ahead**	“Work on being ahead of things – think for myself what is needed”
	**Demonstrate self-awareness**	“Take a few extra seconds when I am called to an acutely ill patient” “Take a step back as a team leader not to lose the overview”
**Decision making**	**Identify options**	“Think of why I do things and reflect on what is best to do now or later”
	**Choosing and** **implementing decisions**	“Communicate what we think, see and do”
	**Reassess decisions**	“Do a sum up to maintain the overview” “Time out for everyone to be able to follow what is going on”
**Leadership**	**Plan and prepare**	“Use a systematic approach” “Do things in the right order”
	**Prioritise**	“Be better at providing the vital parameters in the right order” “Sort the information and structure the orders in a prioritised row of order”
	**Identify and use resources**	“Delegate tasks, could one take care of the relatives?” “Be aware of not sending all out of the room to fetch things” “Offer my help
	**Use authority**	“Would make an overview of the situation and say things aloud” “Be aware of my role as a consultant. How much should I do?” “I have heard what you said, but prioritise something else now
	**Provide and maintain standards**	I will secure documentation during the case
**Teamwork**	**Exchange information**	“Express what makes me wonder, so the other team members can come up with suggestions” “Speak loud and clear, and use closed loops” “Ask more questions and involve the team members – what do they think about their experiences” “Use the ABCDE structure for all to hear” “Use ISBAR, it works but often I forget it” “Ask what the team expects from me”
	**Assess competencies**	“Get the person with the right competencies to do the task”
	**Coordinate activities**	“Remember to use the persons coming into the room instead of doing it my-self”
	**Support others**	“Keep calm and get things done” “Let the less experienced nurses do it and support them instead of taking over as I usually do” “Give my colleagues room and see what they need”

### Feasibility of the training intervention

The team members evaluated the simulation and debriefing structure to promote peer feedback positively, and all the responses ranged from 4.6 to 5.0 on the Likert scale. The response rate was 100%. Table 3 shows the results of both the answers provided immediately after training and following a clinical situation.

**Table 3:** Evaluation of the feedback situation

	***N***	**Question 1** I found the situation meaningful	**Question 2** I perceived the debriefing situation safe	**Question 3** The debriefing situation gave me the opportunity to say, what I found most important	**Question 4** The debriefing situation made me reflect on my clinical practice
**After the in situ simulation**	94	4.8	4.9	4.8	4.8
**After a clinical situation**	44	4.6	4.8	4.8	4.7

*N*, number of participants

The initial reactions of the team members to providing interprofessional feedback after the simulations are shown in Table 4. Five themes were identified. The citations indicate that team members found peer feedback useful. This approach allowed them to reflect on their own clinical practice: “We all grow from receiving feedback”, and “we have a common understanding of the situation, and it is OK to ask curious questions”. They expressed that it was meaningful to promote feedback skills to facilitate dialogue and learning in clinical practice. In addition, they realised that feedback had positive implications for patients as well. Overall, the team members also saw the potential for applying their feedback skills in other situations. “It can be done short but has the value of gold in difficult situations”. Table 5 shows some of the citations illustrating both opportunities and barriers for implementation. The team-members expressed a broad range of opportunities created by peer feedback. One important barrier mentioned was time.

**Table 4:** The team members’ initial reactions to providing feedback interprofessionally

Themes	Quotes about feedback
**Personal development**	“It can change my practice” “A month ago, I was truly in doubt if I had done the correct thing” “The feedback was personal so I could use it—not general” “I have learned something that makes it possible to change my practice” “Good to get words on what you are good at and what could be improved to be better in the future” “Difficult to change ones’ habit if we don’t talk about it”
**Interprofessional collaboration**	“It is OK to ask another profession for advice or help” “Younger doctors are often ‘lonely riders’, we don’t know if what we do is meaningful” “Get a common understanding of the situation” “After a situation reflect together on what we could have done differently
**Interprofessional communication**	“It is possible to communicate more between the professions It is OK to say: ‘I wonder, or I am curious’” “It will be easier now I know how to do it
**Attitudes**	“Learning from each other’s good practice” “It is now OK to ask another profession about what happened

**Table 5:** Overall opportunities and barriers to providing feedback

	**Opportunities**	**Barriers**
**Learning**	“Important to understand that it is about learning, not critique” “It is not dangerous” “Feedback is constructive” “Can be used in other situations” “Will be easier the more we use it” “Can be done short, but have the value of gold in difficult situations” “Possible to talk about the things that do not work so well	“Can be difficult if I have been running in and out of the room and not seen what has been going on” “Difficult to find time in clinical practice” “Implementation will be difficult in our ward
**Culture/professions**	“We should grow together” “I did not think that the physicians needed it (nurse)” “Nurses might need it more as we do not have the same medical expertise (nurse)” “Much better than having a lot of questions and disputes” “Better understanding for each other’s tasks and workflow	“It can be difficult to do with some of the doctors”

## Discussion

The team-based in situ simulation scenarios provided insight into team members’ social and cognitive skills. The self-debriefing using a debriefing structure and the use of open-ended questions promoted team-based reflections and facilitated peer feedback skills. The individual learning objectives written by the team members represent their reflections and how they intended to improve their skills. All the participants recognised the positive potential of peer feedback after critical patient situations in real clinical practice. Overall, the training method was found to be feasible.

The data collected before the intervention made it obvious that one of the main challenges was related to being a newly educated nurse or physician and being able to collaborate in challenging situations without knowing other team members’ competencies. In addition, more senior physicians were not aware of these difficulties. The challenge of coming from the academic environment to working as a nurse or physician in a busy clinical environment is in accordance with the literature [20–22]. The workshop participants mentioned the need for a short defusing and reflection on what could be improved after taking care of a critically ill patient. They expressed the need for interprofessional feedback after a critical situation.

The intervention consisted of several initiatives, which were intended to prepare the team members as well as possible for the simulation sessions and debriefings. Our intention was to create a situation that was as close to the clinical situation as possible to facilitate the use of peer feedback in the clinical setting. Hence, we chose to conduct in situ simulations and to make use of the department’s own equipment/drugs and emergency notifications to increase the applicability of feedback to real-world situations.

In situ simulations to train medical expertise as well as social and cognitive skills are increasingly used in the training of emergency teams, such as resuscitation and trauma, as well as for training ward teams in identifying and caring for deteriorating patients [23–26]. Usually, a debriefing is conducted after the simulation by a trained facilitator. In this study, however, we used in situ simulation and a debriefing structure to train team members to provide peer feedback to each other independent of their profession and across hierarchies to stimulate team reflexivity. In the description phase, each participant provides an important piece to the puzzle as not all staff members are present at the same time When the team has a common understanding of the situation, it is possible to clarify potential misunderstandings and missing information. In the analysis phase, the use of open-ended question and being curious about the frames behind the actions facilitate team reflexivity. The use of this type of questions is in line with the work of Schein [27]. The team member will then know exactly what to repeat to achieve good clinical practice; furthermore, the feedback provider reflects on what is good practice in this situation and what actions to apply by them themselves. Our findings agree with the conceptual framework for team reflexivity described by Schmutz and Eppich [5]. In our study, we focused on post action team reflexivity, which facilitated the development of a shared mental model. The verbalisation of learning objectives about teamwork, leadership, situation awareness and decision-making illustrate the team members’ ability to reflect on action in the simulations. The collective learning creates an opportunity to improve future work and enhance patient care.

Based on the evaluations of the training and the use of peer feedback in the clinical setting as well as team members’ initial reactions and positive reflections on peer feedback as a method, we found the learning method feasible. It made the team members reflect on both the medical treatment and how the social and cognitive skills could support them in the work. Our findings are in line with the recommendations of including focused discussions after both routine and nonroutine events. Clinical debriefing provides unique possibilities for improving teamwork and supporting the reflection of all team members [28–30]. It can be conducted immediately in small groups after a clinical situation. Other ways to facilitate clinical debriefings and improve reflections on action have been published; one example is the TALK framework, which consists of 4 elements: target, analysis, learning and key actions [31]^.^ This concept was initially used immediately after surgical operations, first supported by a local facilitator and then led by the team itself [32]. However, in this project, the purpose was to encourage peer feedback after critical situations, which cannot be planned for.

In the training, we managed to establish a safe learning environment as judged by the team members’ reflections and reactions. The question is if it is just as easily established among all staff members, of which some might not have participated in the training. One of the barriers mentioned for implementation was that it can be difficult to provide feedback to some team members. Psychological safety is strongly related to peer support. According to studies by Edmondson, you may feel more confident speaking up if you have a good understanding of what is expected of you on the job and encouraged by your colleagues [13]. Psychological safety occurs when people speak up, offer ideas and ask questions without fear of being punished or embarrassed. The team should ideally feel safe in testing their thoughts—frames—to identify individuals’ understanding of which actions can be used to elicit a given result. The metaphor of a “safe container” for learning was introduced for learning in briefings before simulations but is just as important for debriefing and feedback [33, 34]. The learning process is an experience-based and social process taking place in the workplace, and feedback is an essential part of this process [35]. However, feedback is a complex process, and there is no simple recipe for receiving and delivering feedback. The interplay between fear of negative feedback and one’s confidence plays a major role in willingness to give and accept feedback [36]; arguably, aspects that are not easily talked about, e.g., trust and feelings, must be considered [37].

The training we proposed might make it easier to use emergent learning opportunities and create momentum for learning in the clinical environment instead of taking learners out of the clinic. This finding is in agreement with a previously proposed framework for emerging learning opportunities in clinical situations and how the use of feedback can promote workplace-based learning [37].

The team members’ initial reactions to providing interprofessional feedback were positive, and examples of such reactions illustrate possible positive learning outcomes at both the individual and team levels. In addition, the team members saw the potential benefit for the patient. They see more opportunities than barriers for conducting interprofessional feedback e.g. “it is about learning, not critique” and “we should grow together”. They have realistic thoughts about having the time to do so. It can be difficult to find the time if some of the team members must attend to other patients. The heads of the department supported the project and made it possible for staff to attend the training. Maintaining interest and supporting the project are important if the implementation is to be successful.

## Discussion of the methods and reflexivity

To avoid bias, none of the facilitators delivering the simulations were part of the project group; hence, the authors did not have any influence on the evaluations. The facilitators are part of a faculty development program in CAMES, and they were all briefed about the project and supported by a course manual.

We found thorough preintervention data collection to be useful for developing an intervention to match the learning needs of participants and conduct simulations to illustrate common frequent patient cases in the ward. Previous team training in the initial treatment of deteriorating patients was conducted in the wards; however, we did not ask about this experience on the evaluation form.

We based our debriefing practice on the methods described in the literature, as we found many parallels between debriefing after simulation and feedback in the clinical setting. The three phases described by Steinwachs and the debriefing constructs by Rudoph et al., including the gaps and peaks model, informed our efforts to enhance feedback in the clinical setting [10, 15, 38, 39].

We chose to let the interprofessional feedback take place without interference from the instructor based on inspiration from the literature. For example, in previous published work, interprofessional team debriefings without an instructor were used after a simulated crisis scenario on targeted crisis resource management content; the authors found that this approach created opportunities for participant learning reflection and suggested that it can be used in clinical practice [40]. Our study indicate that it is possible to train team reflexivity and providing peer feedback after in situ simulations, and if these skills can be applied after clinical situations present, it would potentially enhance patient care.

Integrating feedback and debriefing traditions has been suggested, as both strategies stimulate improvement in performance through learning conversations [41]. We believe our effort to use simulation for training peer feedback skills is one step in that direction.

The context-mechanism-outcome hypothesis can be used to evaluate a program. Our hypothesis was that interprofessional, in situ team-based simulations could encourage teams to engage in reflective conversations in the real-world clinical practice. The outcome of the study is that team-members, at a metacognitive level, saw the opportunities of peer feedback. The individual learning objectives represent their reflections on how they intended to improve their skills. Hence, our study indicates that in this specific context and for these team-members, the mechanism was appropriate.

## Conclusion

The interprofessional in situ simulations with self-debriefing using a debriefing structure promoted team reflexivity and team members’ interprofessional feedback skills. The team members’ reactions to peer feedback were positive, and based on their reflections, peer feedback has the potential to improve both individual and team skills as well as patient treatment. Our method for using feedback to train team members in providing feedback is feasible and could be used in other situations and specialties.

## Data Availability

The data are stored under secure conditions and are available from the corresponding author upon reasonable request.
